# Evaluation as institution: a contractarian argument for needs-based economic evaluation

**DOI:** 10.1186/s12910-018-0294-1

**Published:** 2018-06-13

**Authors:** Wolf H. Rogowski

**Affiliations:** 1 Department of Health Care Management, Institute of Public Health and Nursing Research, Health Sciences, University of Bremen, Bremen, Germany; 2 Helmholtz Zentrum München, German Research Center for Environmental Health (GmbH), Ingolstädter Landstraße 1, 85764 Neuherberg, Germany

**Keywords:** Normative economics, Ethics of health economic evaluation, Constitution economics, Ethical contract theory, Contractarianism, Contractualism

## Abstract

**Background:**

There is a gap between health economic evaluation methods and the value judgments of coverage decision makers, at least in Germany. Measuring preference satisfaction has been claimed to be inappropriate for allocating health care resources, e.g. because it disregards medical need. The existing methods oriented at medical need have been claimed to disregard non-consequentialist fairness concerns. The aim of this article is to propose a new, contractarian argument for justifying needs-based economic evaluation. It is based on consent rather than maximization of some impersonal unit of value to accommodate the fairness concerns.

**Main text:**

This conceptual paper draws upon contractarian ethics and constitution economics to show how economic evaluation can be viewed as an institution to overcome societal conflicts in the allocation of scarce health care resources. For this, the problem of allocating scarce health care resources in a society is reconstructed as a social dilemma.

Both disadvantaged patients and affluent healthy individuals can be argued to share interests in a societal contract to provide technologies which ameliorate medical need, based on progressive funding. The use of needs-based economic evaluation methods for coverage determination can be interpreted as institutions for conflict resolution as far as they use consented criteria to ensure the social contract’s sustainability and avoid implicit rationing or unaffordable contribution rates. This justifies the use of needs-based evaluation methods by Pareto-superiority and consent (rather than by some needs-based value function per se).

**Conclusion:**

The view of economic evaluation presented here may help account for fairness concerns in the further development of evaluation methods. This is because it directs the attention away from determining some unit of value to be maximized towards determining those persons who are most likely not to consent and meeting their concerns. Following this direction in methods development is likely to increase the acceptability of health economic evaluation by decision makers.

## Background

The economic methods to address the sensitive topic of evaluating new healthcare technologies are under ethical debate. This debate is highly relevant for coverage decision practice: to be useful as decision aids, economic evaluations should not only be embedded into a consistent theory but their underlying value judgments also need to correspond with those of the relevant stakeholders and the legal framework within which they operate. A well-known early example of resistance to the use of economic methods in healthcare resources allocation is the Oregon Health Plan’s failed initiative to prioritize health services on the basis of a simple cost-effectiveness calculation only [1, 2]. A more recent example is the debate around the most appropriate use of cost-effectiveness analysis methods in the German healthcare system and the decision maker’s rejection of methods proposed by the health economic community [3–5]: On the one hand, the German principles of coverage decision making are to a large extent based on the notion of medical need (see Needs-based economic evaluation) so that the mainstream evaluation methods based on individual preferences and willingness to pay (see Preference-based economic evaluation) were not considered acceptable. On the other hand, needs-based evaluation methods oriented at a societal decision maker who aims at maximizing health outcomes (see Societal decision maker maximizing health) were also considered unacceptable because, rather than at the idea of maximizing consequences, the German regulatory framework as well as the ethical/moral commitments in Germany seem to be strongly influenced by Kantian ethics and oriented at meeting individual rights [6].

### Preference-based economic evaluation

The standard methods of economic evaluation rest on fairly weak value judgments, i.e. value judgments which require few ethical assumptions and which are assumed to receive wide-spread support: They hold that different resource allocations should be ranked only with regard to the preferences of the individuals affected by it. This incorporates the assumption that individuals are the best judges of their welfare. A widely accepted value judgment of economic efficiency in this context is the Pareto principle which states that if at least one individual prefers resource allocation A over B and all others rank A at least as high as B, then A should be ranked higher than B ([7, 8], p. 2ff.).

Following these value judgments, health economic evaluation involves assessing the preferences and willingness to pay (WTP) of those who pay for health care to determine whether a new technology is included into the benefit basket or not. Given that future health is uncertain, the question of which health technologies should be covered by health care payers could be answered in analogy to the voluntary market choices of insurance contracts with or without the specific technology. Based on a balanced description of the risk of the condition to be cured and the costs and effects of the cure, the preferences revealed by market choices can be simulated by means of cost-benefit analysis (CBA) ([9], p. 11 ff) and coverage granted or rejected accordingly.

Despite the claim that the embedded value judgments command widespread support, using the standard methods for evaluating new medical technology has been subject to ethical criticism for several reasons. For example, individual WTP is not generally considered an acceptable criterion for allocating healthcare resources because of its dependence on ability to pay. This conflicts with frequently held moral intuitions that healthcare should be allocated independent of individual wealth on the basis of criteria such as medical need. Also, health policy typically includes distributional questions and concerns for disadvantaged groups in society who on average bear a larger share of the total burden of disease. Given that generally, the Pareto criterion is indifferent to distribution, it can be considered unsuitable for health policy decisions [10, 11]. Also the practice of coverage decisions appears to follow these critical lines: all health care systems refer to medical need in their decision processes [12] and cost-benefit analysis and WTP measurement only play a minor role in economic evaluation guidelines specified by health technology assessment bodies.^1^ Furthermore, health care is typically not left to the voluntary choice of private health insurance but financed within obligatory tax-funded or statutory health insurance systems with income-related contributions ([13], p. 264ff).

### Needs-based economic evaluation

The most influential response to this criticism was the development of new evaluation methods which incorporate further value judgments. Rather than considering individual preferences only, these health economic evaluation methods aim at measuring health need and its amelioration. “Need” and “needs-based evaluation” can be interpreted differently.

A health care intervention can be said to meet a medical need if it is reasonable and effective, on evidence-based standards of medical practice, for the treatment of some disease, injury or disability [14]. This combines two notions of need. First, following the understanding of “health-related need”, it relates to a gap between patients’ actual health state and a normal health state defined by some standard ([15], p.131 ff.). One standard which is shared by ethicists like Daniels is Boorse’s understanding of health as normal functioning or the absence of pathology ([16], p. 28 f., [17]). This is also the understanding in German Social Court ruling since a decision by the Prussian Higher Administrative Court from 1898 ([18], p. 147 f.). Second, it can be understood as “care need”, i.e. a situation where an intervention exists for which there is sufficient evidence that it can ameliorate health-related need ([15], p. 131ff.). That an intervention meets a medical need, that it is clinically effective and that it produces health gains can then be seen as interchangeable. It has to be noted that this excludes enhancement, i.e. biomedical interventions which improve an individual’s functioning beyond the normal range. This corresponds with a widely held ethical position that treatment should be prioritized over enhancement [14].

Also regarding evidence of effectiveness, different standards can be applied, and in a situation where health care resources are not sufficient to meet all needs, evidence of effectiveness is not a sufficient needs-based criterion for coverage determination [19]. Therefore, it has additionally been claimed that needs-based allocation of health care resources involves covering health technologies only if the cost for the health gain they incur is considered acceptable [20]. One candidate for a benchmark of cost-effectiveness is the cost-effectiveness of health technologies displaced elsewhere in the system [21]. Also, there are proposals to integrate considerations of health-related need, care need and cost-effectiveness by means of severity weights for health states [22]. Finally, needs-based evaluation could also be interpreted as evaluation based on a weighted index which combines different concepts of need [23, 24].

Developing and justifying specific methodological standards of needs-based economic evaluation is beyond the scope of this article. Also, the question of how the evidence generated by these methods is appraised and which claims can be derived from them cannot be addressed here. The relevant distinction for the argument made here just differentiates between preference-based and needs-based evaluation. Preference-based evaluation is understood as an evaluation which involves market-oriented decision processes facilitating individual, subjective choice. Needs-based evaluation is understood as an evaluation which involves political decision processes facilitating collective, evidence-based choices oriented at explicitly defined, objectively verifiable standards. Determining such standards of need and needs-based allocation are an integral part of needs-based decision making but not of preference-based decision making. This is, because such standards are irrelevant if the individual preferences alone determine the optimal choice.

Besides this difference, allocation of scarce health care resources based on needs-based economic evaluation can be seen as incompatible with Paretian, preference-based evaluation for a second reason: it involves progressive financing with systematic net income transfers from the wealthier to the less wealthy individuals in society. This is because of the negative association between income and burden of disease. There is consistent evidence that the poorer groups in the population are, on average, also those who are more affected by diseases [25, 26]. Theoretically, if health care resource allocation was left to unregulated markets or to purely risk-based insurance contracts, health care could still be funded progressively because wealthier individuals acquire more health care services or better health insurance, despite their lower burden of disease. However, if health care resource allocation is based on medical need and access is equal, the wealthier population groups are paying above and receiving below average. This progressivity is intensified in financing systems based on income-related premiums which is frequently the case ([13], p. 264ff). Also, developing and justifying further details of the public health insurance as well as the type of funding and the extent to which it is or should be progressive is beyond the scope of this article. The important feature here is just that its financing is progressive (i.e. those with higher income or wealth contribute more) which, at first sight, it is not compatible with preference-based, Paretian economic evaluation.

Also the value judgment that rather than modelling individual market choice, health economic evaluation should be seen as a problem of maximizing (equity-adjusted) health subject to resource constraints [11] has been subject to ethical criticism. For example, maximizing health with or without adjustments for fairness preferences has been criticized on the grounds that it neglects that treating individuals fairly is fundamentally different from maximizing something considered valuable. The statement: “more health gain is better” could be challenged by the question: “better for whom”? Surveys which aim at determining severity weights by trading off health gains for different population groups frequently experience protest answers or statements that it is important to treat individuals equitably. This can be interpreted as the non-consequentialist value judgment that fair decision making requires that we view patients as individuals with dignity and claims to healthcare resources rather than viewing clinical situations and patients only as means to the end of producing (equity-adjusted) health [2]. This turns the issue at stake away from the question of which outcome is maximized by the evaluation methods to whether applying the methods and their results can be justified and are acceptable to those affected by it.

### Strategies for justifying needs-based evaluation

In standard economic theory, the use of preference-based evaluation methods and the Pareto criterion are justified on the grounds that decisions about the allocation of public resources should be based on rather weak value judgments which command wide spread support ([8], p. 2ff.). Developing and justifying a content-rich concept of “need” is ethically more demanding than simply asking individuals for their preferences regardless of how they are motivated. Different approaches for ethical justifications exist which include the following three.

#### Societal decision maker maximizing health

One early rationale for using needs-based economic evaluation in the United Kingdom was the concept of a societal decision maker assigned with the task of maximizing health outcomes. Rather than a fully developed ethical theory, the concept assumed that the health policy decision makers specified “meeting health needs”, or equivalent, “producing health” as the relevant objective function. Economic evaluation is seen as a type of analysis commissioned by this decision maker to maximize this function [27].

This concept has the advantage that it fits well with welfare economists’ predisposition to analyze different social states in terms of quantifiable social welfare functions. However, due to the lack of an elaborated ethical theory, it leaves many questions unanswered, like, for example, the question of how “health” should be specified. Also, this concept is subject to the criticism above that, at least in countries like Germany, the aim of health care is not adequately characterized by maximizing aggregated health outcome.

#### Contractualist aim of meeting health needs fairly

Probably the best known ethical theory of health care justice is Daniels’ expansion of Rawls’ justice as fairness to the health care sector. He starts with a notion of medical need – healthcare is special because treating disease and disability restores normal species functioning and normal functioning is necessary for individuals to access the full range of opportunities open to them. In consequence, needs-based considerations are highly relevant for deciding which health needs to meet if resources are insufficient to meet all of them. However, Daniels rejects establishing one specific evaluation methodology. Even if he suggests drawing upon needs-based economic evaluation methods like those proposed by Nord [22], he insists that ultimately, reasonable people will disagree about how to prioritize health care. Therefore, the evaluation of new medical technologies should take place within a fair process which is accountable for the reasonableness of the decisions [28].

Daniels’ appeal “to reasons and principles that are accepted as relevant by people who are disposed to finding terms of cooperation that are mutually justifiable” [28] illustrates his commitment to contractualism. In modern ethical contract theory, two schools of thought can be distinguished. Both schools of thought start with the premise that societal collaboration is highly beneficial so that the members of modern societies have an interest in securing this collaboration by means of a social contract. However, contractarianism follows the Hobbesian line of thought and assumes that individuals are primarily self-interested. They act morally and consent to government authority only as far as a rational assessment of the situation reveals that this maximizes their self-interest. Contractualism, instead, follows the Kantian line of thought and holds that rationality requires that we respect persons. This involves that moral principles are such that they can be justified to each person [29].

Daniels’ theory of just health is widely supported and the use of needs-based economic evaluation methods by the English and Welsh National Institute of Health and Care Excellence (NICE) has been associated with his theory ([16, 30], p., 112). Nevertheless, its contractualist ethical assumptions may be as difficult to accept for an economist who is looking for weak ethical value judgments as the idea of evaluating need and its amelioration.

#### Other justifications

There are other arguments to support economic evaluation methods oriented at alleviating need rather than at simulating market choice. These may not necessarily be combined with contractualist or contractarian lines of argumentation. For example, the ethical considerations underlying Amartya Sen’s Capability Approach provide one influential example [31–33]. Also, from a communitarian perspective, it has been proposed to re-focus health economic thinking towards community values which may also promote health economic evaluation based on medical need [34]. Given the practical importance of medical need in the evaluation of new medical technologies, further development of these different ethical considerations is welcome.

This paper explores an alternative way which may address the fairness concerns, be consistent with the standard economic methodology and justify health economic evaluation methods measuring medical need rather than measuring preference satisfaction. To achieve this, it follows a constitution economics view which is closely linked to contractarian ethics. From a contractarian perspective, societal institutions like a statutory health insurance are justified if all members of society can, at least hypothetically, consent to it because it provides an advantage to them [29]. Rather than a problem of maximizing the utility in terms of WTP or of maximizing some outcome, health economic evaluation is analyzed as an instrument for resolving conflicts of coverage decisions in a manner all involved stakeholders can consent to.

The paper starts with briefly introducing contractarian order ethics. It is then illustrated how the institution of an obligatory statutory health insurance with individual claims to funding medically effective healthcare can be understood within this ethical framework. The use of needs-based health economic evaluation is interpreted as an institution to overcome co-ordination problems in such statutory health insurance systems, to the benefit of all affected parties. The final paragraph briefly discusses implications of this view for health economic evaluation as well as the relation of this article to previous work and concludes.

## A contractarian argument for needs-based economic evaluation

The application of contractarian considerations on economic problems is an approach which is strongly influenced by James Buchanan [35, 36] and which is explained and applied in the subsequent sections.

### Contractarian order ethics

Buchanan proposes making a distinction between choices *within* rules and choices *of* rules. The choice of acquiring a health technology on the market which is modelled by CBA is a choice within rules. This article addresses the choice of rules.

Rules are enforceable constraints to the freedom of individuals. Therefore, the question arises why an individual should voluntarily choose or consent to rules at all. Buchanan’s answer is that, while rules decrease the range of actions available to individuals, they can at the same time increase the range of options for cooperation because they decrease the costs associated with preparing, negotiating and implementing these co-operations ([24, 37], p.).

The order ethical approach to contractarianism developed by German economic ethicists like Karl Homann and Christoph Lütge largely builds on Buchanan’s ideas but proposes using them to support the implementation of ethical norms. It claims that typically, ethical considerations can be translated into aims of self-interested individuals, for example, if the time horizon of the self-interested calculation is extended or if forgone gains from cooperation are considered. Ethical problems in economic interaction are assumed to result in an unintended manner from individual behavior following suboptimal incentive structures. Rather than blaming individuals for their unethical intentions and behavior and demanding better individual comportment, order ethics suggests changing the incentive structure (the “order”) in which the individuals operate. Order ethicists hold that, in a pluralist society, ethical norms in economic interaction can only be implemented if the affected stakeholders recognize that they actually forego gains from cooperation and if they agree on rules that channel their behavior in a way that allows the gains from cooperation to be realized ([38], p. 3ff.).

In more detail, order ethics assumes that human interaction generally features characteristics of social dilemma structures ([38], p. 93ff.): there are always both mutual and conflicting interests, and conflicting interests may impede productive collaboration. This may occur because the result of a cooperative activity cannot be achieved by one cooperating party on its own. In the face of conflicting interests, each party has to fear that her efforts towards the collaborative result will be exploited by the other party. Mutually beneficial collaboration is therefore at high risk of failure unless both parties can create stable expectations about the other’s behavior – enforceable rules for their interaction ([37], p. 36ff).

Social dilemma structures can be illustrated with the well-known game theoretic situation of the prisoner’s dilemma (see Table 1): compared with situation (III), the game theoretic players P and S were better off in situation (I). However, both players have incentives to defect. Therefore, benefit maximization leads both players towards situation (III) – an equilibrium in dominant strategies towards a Pareto-inferior social state ([37, 39], p.).

**Table 1:** Dilemma structure

	Physicians (P): Cooperate vs. defect	
Sickness funds (S): Cooperate vs. Defect	(I) P:2, S:2	(II) P:0, S:3
	(IV) P:3, S:0	(III) P:1, S:1

Consider, e.g. the interaction between physicians (P) and sickness funds (S). Both parties have shared interests: that the patients’ disease is cured – for doctors to earn their living, for insurance funds because without medical services there is no reason to have health insurance. On the other hand, they also have conflicting interests: physicians are likely to be interested in unlimited expansion of reimbursed services; health insurance funds in cost containment to keep the contribution rates affordable. Without rules to resolve these conflicts, there is a high risk that collaboration (e.g. reimbursement of a beneficial new health technology to a physician) fails.

Rules which stabilize expectations about how (potential) cooperation partners will act are termed “institutions” and can be analyzed by different theories within New Institution Economics among which also constitutional economics can be subsumed. Besides incentives to deviate from cooperating behavior, it also incorporates the analysis of information deficiencies about the cooperation partners’ motivation, characteristics and actions [40]. In the example above, it would predict that the cooperation would fail because the insurance fund’s ability to control the doctor’s actions is limited. Therefore, moral hazard (if patients are seen as those who request services) or supplier-induced demand (if service provision is seen to be determined by physicians) is likely to induce excessive use of pooled healthcare resources. In modern healthcare systems, multiple institutions have evolved that enable cooperation by overcoming these problems of interactions, e.g. reimbursement rules which provide physicians with stable expectations of income from insurance funds and at the same time limit funding to selected technologies [41].

It has to be noted that the institutions and the associated incentive structures in modern health care systems are highly complex [41, 42]. Therefore, the model of the dilemma structure depicted in Table 1 is unlikely to be found in its simple form in health care practice. Rather than using the term “dilemma structure” as a description of a social situation which fulfils all the characteristics of the stylized game theoretic situation of the prisoners’ dilemma, order ethicists propose using it as a guidance for searching solutions to social conflicts. This guidance may direct the search towards (potentially unnoticed) shared interests and institutions which help improve social co-operation ([37], chapter 6). These institutions are subject to continuous improvement as long as there is room for mutual gains for those who are affected by them [43].

The order ethical approach contains both positive and normative aspects. Positively, it is based on the assumption that moral problems contain (potentially hidden) attributes of a dilemma-like situation so that they can successfully be re-framed as such and new gains from cooperation identified and realized. However, from the fact that the dilemma structure is to be used as a tool to support the search for these gains from cooperation, it becomes visible that the approach relies on implicit normative assumptions: it assumes that individual, self-interested preferences rather than ethical considerations beyond these are relevant for evaluating different social states. And it proposes that rules which lead to Pareto improvements should be identified and implemented. To achieve this, a first task is to elaborate how an ethical problem can be understood as a social dilemma and which new rule might be suited to overcome it.

### Healthcare financing as a social dilemma resolved by institutions

Free market exchange is believed to be a major stimulus to economic growth, and both healthcare and health insurance are included among the goods and services which could be acquired within such voluntary exchange. Nevertheless, all modern economies have developed institutional settings for provision and financing of healthcare services which typically involve obligatory enrolment into statutory health insurance or funding through obligatory taxes. Given that these insurance premiums or taxes are based on income, not on risk like actuarially fair premiums, they are likely to involve income transfers from healthier and wealthier “good risk” individuals to less healthy and wealthy individuals with high-risk of disease. From a contractarian perspective, the question arises why primarily healthy contribution payers should consent to an obligation to fund the healthcare of their sicker fellow citizens rather than insisting on risk-based premiums.

One reason can be that care serving health needs is fundamentally different from goods acquired on markets to satisfy desires. Desires expressed in preferences are unlimited and subjective. In contrast, if health is understood as normal functioning or the absence of pathology ([16], p. 28 f., [17]), the need for physical health is limited and susceptive to objective measurement ([44], p., 109). Different from desires, if basic needs such as the need for health go unsatisfied, then “serious harm of some objective kind will result” [45].

Unmet health needs can induce different kinds of harms. First, they can lead to avoidable disease, disability and premature death. Second, they can decrease the possibility of productive participation in society, e.g. on the labor market. Third, since the need for care in the case of disease or disability features low price-elasticity, it can lead to catastrophic healthcare expenditures. These expenditures can keep or push families below the poverty line, particularly if combined with lower household income due to ill health [46, 47]. Also, they can lead to coping strategies which induce hidden chronic poverty such as selling assets [48].

Unmet medical need and insufficient healthcare financing is also a disadvantage for the healthy citizens for multiple reasons, apart from the fact that some healthy citizens may have sympathy for the sick and egalitarian preferences. E.g. avoidable disease, disability and premature mortality decrease the opportunity for co-operation because productive human capital is forgone [49]. Apart from indirect disadvantages in terms of co-operation forgone, there are also direct disadvantages because increased poverty and deprivation increases the risk of crime and political instability in a society (see quadrant III with payoffs of 1 for both healthy contributors to the sickness fund, S, and patients, P, in Table 1).

In a natural state, the equilibrium in a state of high out of pocket financing of healthcare and political instability is maintained, first, because even if benevolent sickness fund contributors were willing to cooperate with the patients in need of healthcare financing, this is unlikely to be possible in a financially sustainable manner: free health services set incentives for an unlimited number of patients to seek an unlimited scope of services without financial contribution - which can easily exhaust the sickness fund contributor’s capacity to pay (quadrant IV, P:3, S:0). On the other hand, cooperating patient population groups who refrain from exhausting all options to improve their situation, including excess use of unpaid services, crime, or threatening political stability have to fear catastrophic health and healthcare expenditures without any alleviation (quadrant II, P:0, S:3).

An obligatory healthcare financing system by taxes or a statutory health insurance where the burden of healthcare financing is shared by all citizens and therefore reduced to an affordable amount can be seen as an institution to obtain a Pareto-superior social state (quadrant I, P:2, S:2). Consent by the sickness fund contribution payers could be achieved, e.g. by granting individual claims to healthcare financing also for them in case of disease in turn for the obligatory enrolment. Also, it might have to be assured that the obligatory healthcare contributions are kept at a minimum and exclusively spent for the purpose of meeting objective health needs rather than for funding further services which may be preferred by the patients but which are not necessary to avoid or ameliorate harm.

At least for the Bismarck health insurance systems in Europe it is likely that such considerations were relevant for their inception: the creation of the statutory health insurance in Germany by Bismarck was primarily a means to stabilize the state against socialist revolutionary tendencies ([50], p. 15ff). Rather than a company with the primary aim to fund services which meet the preferences of the contribution payers or a government initiative to maximize some good of social value such as health, statutory health insurance may therefore be appropriately analyzed as an institution evolving from a social contract to meet explicitly determined claims for reimbursement to alleviate need in exchange for social stability, at the benefit of both patients and healthy contribution payers.

### Needs-based economic evaluation from a contractarian perspective

An important element in such a social contract is a further specification of “medical need” and under which circumstances a service to alleviate medical need is to be publicly funded. This is of particular importance because easily, conflicts can arise between the contract partners in the face of incentives for over-provision of health services and potentially differing definitions of “need”, uncertainty about a technology’s effectiveness in alleviating need, and difficulties in monitoring the health care providers’ actions.

One highly effective institutional arrangement to resolve conflicts is the use of pre-specified, objective evaluation criteria because the question of whether or not a shared aim was met can be disentangled from individual perceptions and positions [51]. This may explain why objective scientific evidence that a medical intervention is suitable to improve health outcomes has become a key criterion of healthcare coverage decisions worldwide [52]. Also, the institutional design of the decision process can serve this aim: transparency and participation of key stakeholders can be seen as a means to ensure that the consensus is maintained and interests of both parties are appropriately taken into account [41, 53].

Over time, new conflicts may arise and the environment may change so that institutions have to be continuously adapted. In particular, due to medical progress, the number of situations which can be assigned a “medical need” has expanded because new diseases and syndromes have been defined. Also, the number and costs of technologies available for their alleviation have increased. Given the extraordinary ability of medical technology to consume resources ([54], p., 434), escalating health care costs may threaten the sustainability of the societal contract for health care coverage. Therefore, solutions to secure its financial sustainability and contain costs have become a major challenge of health policy which remains to be resolved.

Following the approach applied here, also this challenge could, for the sake of this contractarian argument, be construed as a dilemma structure. Ignoring costs in coverage decisions and officially granting coverage of all services with evidence of effectiveness may then be seen as a Pareto-inferior situation with unrealized gains from cooperation to various stakeholders. This is, e.g. because in the face of cost pressures, such an approach may lead to implicit rationing which causes stress and the risk of litigation for physicians [55]. Also, using evidence as a criterion for cost containment increases the expected development costs for manufacturers [19] and unpredictable ad-hoc policy measures of cost containment further increases their uncertainty of research and development decisions. Also, particularly those patient groups with lower socio-economic status which benefit most from the social contract are likely to be disadvantaged by implicit rationing [56]; and in a scenario of full coverage of all technologies, affordability of contribution payments may be most problematic for this population group.

Complementing the existing, evidence-based coverage criteria by pre-specified, economic evaluation methods which incorporate objective measures of alleviated health needs may therefore be seen as a valuable amendment to the differentiated set of institutions already established to implement the societal contract for health care coverage, at the benefit of all contracting partners.

As an example, decreased expected transaction costs of getting new products reimbursed and decreased research and development uncertainty may make more innovations which would be of sufficient priority for healthcare coverage also profitable from a manufacturer perspective. For patients, it may improve equitable access to most needed care while at the same time providing a means to facilitate cost containment or, at least, controlled budget expenditure for payers. Also, clarity about which services have to be publicly covered creates stable expectations about which services would have to be funded by complementary private insurance if desired.

Even those patients who cannot afford private health insurance can expect to benefit from a decision rule which restricts claims to public coverage of selected innovations: in the long run, the range of services available to them is likely to be larger than in the case of a rule which determines coverage for all patients without allowing for complementary private health insurance. This is because, to ensure affordability, such an obligatory universal coverage scheme is likely to deny coverage to some services which incur high costs at low effects due to high (patent monopoly) product prices and therefore hinder their introduction even if there would have been wealthier individuals who would have the willingness and ability to pay for them. However, had they been introduced, after patent expiry, their cost-effectiveness may have met the benchmark also for public coverage.

## Discussion

The use of individual utility and willingness to pay as criteria for evaluating new medical technologies has been criticized on the grounds that health care should be allocated independent of wealth on the basis of objective criteria incorporating medical need. This article proposes a contractarian approach to address this criticism: the use of economic evaluation in coverage decision processes can be seen as an institutional amendment to implement a social contract of covering health care to alleviate medical need. Given that its justification is based on (hypothetical) consent because benefit arises to all conflicting parties, this view only requires the rather weak value judgments of individual preferences and Pareto efficiency. However, evaluation methods based on objective and interpersonally comparable measures associated with health need are likely to be more acceptable and useful than methods directly based on individual preferences and WTP. While an economic analysis of hypothetical choices within the rules of the market typically promote approaches of simulating market decisions, this analysis of a hypothetical choice of (economic evaluation) rules may exceed beyond tools for measuring individual preferences to any measure the involved stakeholders can agree upon. Therefore, this institutional view also provides a new justification for needs-based evaluation. Given that this view can be associated with a contract ethical basis of acceptability and consent rather than a consequentialist basis of maximizing some favored unit of outcome, it may provide a reply to the fairness arguments against health economic evaluation methods from a non-consequentialist view (e.g. [2]).

Pursuing an order ethical analysis involves theoretical and empirical activities. Theoretically, it involves analyzing moral problems in a way that allows us to detect potentially Pareto-improving rules. In reality, no regulatory order has ever been built on full consensus – e.g. because the population is dynamic, and because obtaining full consensus is very costly. Therefore, consensus needs to remain subject to approximation. One method to approximate consensus which can be used as an alternative is to assess a rule’s generalizability, similar to Immanuel Kant’s postulation that legitimate principles are those every individual could have consented to ([37], p. 186ff). As an empirical activity, to ensure that such a modelled consent is a realistic approximation, it needs to be complemented by political discourse and participation ([37], p. 186ff). This article could only provide a theoretical analysis. The complementary empirical investigation of incentives and preferences of the involved stakeholders was beyond the scope of this article.

### Implications for health economic evaluation

The idea that economic evaluation is a tool for conflict resolution rather than a tool for maximizing some type of outcomes may increase its acceptability to decision makers. At least in Germany, coverage determination is based on individual claims to health services which are to be met equitably, rather than any idea of maximizing utility or valued health outcomes [57]. The decision maker’s rejection of the methods for health economic evaluation proposed by the health economic community in Germany [3–5] may be due to the fact that such a view based on claims and universal acceptability which is consistent with the contractarian justification presented here is not sufficiently incorporated in existing evaluation methods. However, to achieve this acceptability, this view would also need to be incorporated into the economic evaluation methods.

This consensus-oriented view may guide the development of evaluation methods away from attempts to optimize utility measurement or an engineering approach to outcome maximization towards integrating fairness concerns. Further applied work is needed to explore the feasibility of this approach. A first step to achieve this may be to search for stakeholders who might be most likely not to consent. As an example, the contribution payers’ consent to health maximizing evaluation methods is less likely if it puts them at a systematic disadvantage. This can be, e.g. because they are already suffering from a lethal condition so that potential future health is less relevant; because their future health is unlikely to result from the play of chance due to a hereditary disorder; or because they are disabled. A second step might be to further develop the evaluation methodology or the exceptions in a way that these groups could consent to as well. This research can build on existing work on multi-criteria decision making [58] but should appropriately respond to the critique of consequentialist value maximization [2]. “Efficiency” here does not necessarily depend on whether one value like (equity-adjusted) health is consistently maximized. Instead, “efficiency” involves that the decision rule can find consent. This also implies that elements may be included which are inconsistent from a consequentialist output maximizing perspective like “value all quality-adjusted life years gained by disabled and non-disabled patients equal” if this improves the acceptability of the evaluation methods.

As described at the end of the methods section, the development of tools for conflict resolution that citizens can consent to also involves political participation and discourse. Evaluation methods might be more likely to find consent if the stakeholders understand their normative basis, can agree it is a relevant basis for resolving the conflicts associated with coverage determination, and feel that all aspects important to them are incorporated in a valid and reliable manner. Given that population-based preference elicitation provides evidence of mean values but not of consented values, deliberation in smaller groups might be an approach to achieve this.

Finally, successfully developing and introducing new (economic) evaluation methods involves analyzing how the existing processes were able to overcome dilemma structures in the past. This is necessary to design evaluation methods and processes in a way suitable to improve conflict resolution. As an example, from a consequentialist perspective, it can be claimed that expected cost-effectiveness rather than strength of the evidence should be used to determine coverage [59]. However, appraising the strength of the clinical evidence is a key element in most coverage decision processes, potentially because weak evidence can easily be biased in favor of a new intervention. Likewise, those clinicians who are the most experienced specialists to appraise the new technology may not be neutral but may have personal interests in coverage. Proposals to promote the use of economic evaluation should include answers to the question of how economic criteria can be used in decision making without increasing the risk of bias. Managed entry agreements allow the objective measurement of costs and outcomes alongside their use [60, 61] and may hence be a starting point for such proposals.

It has to be noted that this view leaves room for various different approaches to economic evaluation. And, after all, it might not lead to drastic changes in decision methods and practice. For example, in a country where utilitarianism has been very influential, many stakeholders appear to accept an outcome maximizing decision rule; in the appraisal processes of the English National Institute for Health and Care Excellence, some equity constraints are already applied and the institute’s citizens’ committees correspond with the political discourse and participation necessary to achieve consensus. However, if this is the case, this may rather confirm than refute the validity of the argument made in this paper – as it indicates that economic evaluation may already have been used as a tool for conflict resolution rather than for outcome maximization.

### Relation of this article to the existing literature

There is a large body of literature on health economic evaluation, coverage decision criteria and the value of health insurance which can only be addressed very briefly here.

The value of health insurance has been analyzed from a welfarist economic perspective by various authors. Nyman distinguishes three periods of economic thinking about this topic. In a first period, a positive value provided by health insurance has been estimated, based on the idea that it satisfies demand for certainty. In a second period, potential welfare losses due to moral hazard were at the core of the analysis. In a third period until to date, there is a general notion of positive welfare gains, also, because health insurance involves income transfers which reach patients in a situation where their WTP for medical services is extraordinarily high [62]. The analysis presented here deviates from these analyses because of its (constitutional) economics perspective rather than the perspective of individual, market-like choice of a health insurance contract.

Also, the value of social health insurance (SHI) has been analyzed from a welfarist economic perspective. Mandatory SHI has been considered efficiency-increasing in a Paretian view, for example, if it eliminates failures of private health insurance markets associated with asymmetric information or if altruistic rich members are willing to subsidize the provision of health care to the poor. Including public choice considerations, mandatory SHI may also be a vehicle for politicians to gain votes in a setting where redistributive effects may remain disclosed to the net payers [63]. The analysis presented here also analyzes the value of SHI and using social dilemmas, it draws upon a model used in public choice. However, it uses this model as a tool for detecting unrevealed gains from cooperation ([38], p.93ff.) to make a normative argument rather than a positive analysis. Also, the public choice concept is applied to the choice of health economic evaluation methods.

Furthermore, it has also been argued that needs-based rules for coverage determination and compulsory health insurance are actually just a means to handle uncertainty about one’s own future health and the services to be covered by a health insurance contract [14]. However, this argument does not explain why individuals should consent to one national social or government health insurance which is progressively funded. There could be, for example, several financing schemes like in Germany where basically, the healthier and more affluent part of the population is covered by private and the less healthy and affluent part covered by the statutory health insurance. Also, it does not explain explicit means of progressive financing based on taxes or income-related social insurance contributions.

Also Culyer assessed the role of externalities in health and demonstrated that these can be seen as an argument for publicly provided health care. Also, he implied that this involves political processes within which methods for determining health status are to be determined as well as the “engineering objective” i.e. whether total health is to be maximized or e.g. some minimum is to be preserved [64]. This article incorporates the idea of externalities and the idea of political processes to determine the relevant evaluation measures. But rather than promoting a consequentialist strategy of measuring efficiency in terms of an axiology and a related objective function, it argues for efficiency in terms of a rule which can find consent. Even if this may include measures of health gain, and even if in a utilitarian country, a health maximizing rule may find consent, it is more likely that the decision rule also contains elements which are inconsistent with such a strategy of consequentialist maximization.

Also without this close link to economics, contractarian arguments in favor of robust social institutions caring for the sick and disabled in modern societies have been made [65]. However, these were made on the positive grounds that even if only strategic reasons were taken into consideration, policy makers would establish these institutions because they realize that disease and disability might also hit them and people they know. This is because, it could be argued that the sick and disabled are not able to offer attractive gains from co-operation or, at least, to destabilize society. In the argument of this article, it was assumed that they were able to do so, with a reference to Bismarck’s reforms which established the first SHI.

This article only addressed contractarian considerations regarding needs-based economic evaluation methods. As mentioned in the background section, there also is a large body of contractualist literature on health care coverage decision following Rawls and Daniels, in particular Daniels’ needs-based account of just health [16] and his framework of accountability for reasonableness [16]. While Daniels refrains from proposing evaluation methods, this analysis suggests that a further specification of evaluation methods may not only be efficient from a contractarian perspective but also reasonable from a contractualist one. Therefore, there is a need for further work justifying specific needs-based economic evaluation methods from a contractualist perspective. Also, given the importance of needs-based economic evaluation and its unclear normative basis, further work from other perspectives like communitarianism or the capability approach would be desirable.

## Conclusions

The existing normative theories of health economic evaluation are either oriented at hypothetical market choices and WTP which disregards the wide spread notion that coverage decisions should be oriented at medical need; or, they are based on explicit ethical considerations (typically of medical need) and promote the consequentialist maximization of (equity-adjusted) health which disregard non-consequentialist fairness concerns. This article suggests an alternative view: it proposes to view economic evaluations as institutions to overcome conflicts associated with coverage decisions. Evaluation methods based on medical need are argued to be more widely acceptable and therefore better suited for conflict resolution than methods based on hypothetical market choices and WTP. Nevertheless, they are consistent with traditional welfare economics which is oriented at preferences rather than needs because conflict resolution leads to Pareto improvements. This view may promote the integration of fairness concerns into the development of health economic evaluation methods and lead to an evaluation practice which is more acceptable for the variety of stakeholders who are potentially affected by it.

## Abbreviations

CBA: Cost-benefit analysis, i.e. health economic evaluation method which measures benefit in terms of willingness to pay (rather than, for example, life years gained)
SHI: Social health insurance, i.e. a form of health insurance which is typically mandatory and funded largely by income-based contributions of employees and employers
WTP: Willingness to pay

## Footnotes

1 See: http://www.ispor.org/peguidelines/index.asp, categories “Preferred analytical technique” and “Preferred coutcome measure”, accessed on Aug 28th, 2017.
